# Clinical Use of Melatonin as a Cross-Sectional Modulator of Oxidative Stress Across the Perinatal Continuum: From Placental Dysfunction to Neonatal Disease

**DOI:** 10.3390/nu18172817

**Published:** 2026-08-28

**Authors:** Francesco Cipriani, Elisa Furlan, Luisa Lonoce, Elisa Laschi, Michele Minerva, Lorenzo Perilli, Federica Lotti, Salvatore Grosso

**Affiliations:** 1 Clinical Pediatrics, Department of Molecular Medicine and Development, Azienda Ospedaliero-Universitaria Senese, University of Siena, 53100 Siena, Italy; elisa.furlan@student.unisi.it (E.F.); luisa.lonoce@ao-siena.toscana.it (L.L.); elisa.laschi@ao-siena.toscana.it (E.L.); michele.minerva@ao-siena.toscana.it (M.M.); dottorperilli@gmail.com (L.P.); federica.lotti@ao-siena.toscana.it (F.L.); salvatore.grosso@unisi.it (S.G.); 2 Department of Neurosciences, Rehabilitation, Ophthalmology, Genetics, Maternal and Child Health, University of Genoa, 16126 Genoa, Italy; 3 Department of Neuromuscular Diseases, UCL Queen Square Institute of Neurology, London WC1N 3BG, UK

**Keywords:** melatonin, oxidative stress, prematurity, neonatal diseases, hypoxic–ischemic encephalopathy, bronchopulmonary dysplasia, necrotizing enterocolitis, placenta, fetal growth restriction, neuroprotection

## Abstract

Oxidative stress is a central mechanism linking placental dysfunction, fetal injury, and neonatal disease, supporting the concept of a perinatal oxidative stress continuum that begins during abnormal placentation and may persist after birth. This narrative review explores the biological rationale and current experimental and clinical evidence for melatonin as a potential multitarget intervention across this continuum. Melatonin combines direct antioxidant activity with mitochondrial protection, anti-inflammatory and anti-apoptotic effects, and modulation of endogenous antioxidant defenses and redox homeostasis. Evidence from placental, fetal, and neonatal studies suggests potential protective effects in pregnancy complications and in neonatal conditions characterized by oxidative injury, including hypoxic–ischemic encephalopathy, bronchopulmonary dysplasia, necrotizing enterocolitis, neonatal sepsis, and retinopathy of prematurity. The review was informed by a non-systematic search of PubMed/MEDLINE, Scopus, Web of Science and Cochrane Library for relevant literature published up to 2026, including preclinical and clinical studies, systematic reviews, and relevant clinical trial registrations. Overall, although preclinical evidence is encouraging and early clinical studies suggest potential safety and biological activity, translation into clinical practice remains limited by heterogeneous study designs, dosing and timing regimens, and the lack of adequately powered randomized trials. Further research is needed to establish optimal dosing, timing, patient selection, pharmacokinetic–pharmacodynamic relationships, and long-term safety and efficacy.

## 1. Introduction

### 1.1. Oxygen Radical Disease of Neonatology

Oxidative stress (OS) represents a pathophysiological mechanism underlying several fetal and neonatal diseases. It results from an imbalance between the excessive production of free radicals (FR), including reactive oxygen species (ROS) and reactive nitrogen species (RNS), along with the limited capacity of endogenous antioxidant defense systems to neutralize them. During the perinatal period, the imbalance between FR and antioxidant molecules becomes particularly relevant because fetal and neonatal tissues are characterized by high metabolic activity, elevated oxygen consumption and relative immaturity of antioxidant pathways. Consequently, the newborn, and especially the preterm neonate, is highly susceptible to free radical-mediated injury [1]. Early during pregnancy, fetal development and placentation are stages which can be critically affected by OS damage, with possible consequences in peripartum and post-natal life [1]. Accumulating evidence suggests that placental OS is involved in several pregnancy complications, such as preeclampsia, fetal growth restriction (FGR), preterm birth (PTB) and their long-term consequences. Inadequate placental perfusion and ischemia–reperfusion mechanisms induce excessive ROS production, mitochondrial injury, endothelial dysfunction, and inflammatory activation, thereby contributing to increased fetal OS and neonatal disease. This concept supports the hypothesis of a continuous oxidative stress framework extending from placental dysfunction through fetal development to neonatal morbidity, although the causal relationships between these stages remain incompletely established [1,2,3].

Furthermore, the transition from intrauterine to extrauterine life constitutes another critical oxidative event in human physiology. Indeed, fetal development occurs under relatively hypoxic conditions, with arterial oxygen tensions significantly lower than those observed after birth [4]. Therefore, delivery abruptly exposes the newborn to a hyperoxic environment, resulting in a rapid increase in mitochondrial oxidative metabolism and ROS generation. Under physiological conditions, endogenous antioxidant systems counterbalance this oxidative challenge. However, in preterm infants these mechanisms are incompletely developed and are still not calibrated to counterbalance this sudden rise in O2 concentration [4]. Moreover, neonatal tissues, such as cerebral white matter, are still immature and particularly vulnerable to oxidative injury [2,5]. This vulnerability is further amplified by common neonatal intensive care interventions such as oxygen supplementation, mechanical ventilation, blood transfusions, and exposure to inflammatory or infectious stimuli. In parallel, mitochondrial dysfunction, ischemia–reperfusion injury, excitotoxicity, and persistent inflammation contribute to a self-perpetuating cycle of oxidative damage involving multiple organs and systems [1,2].

**Figure 1:**
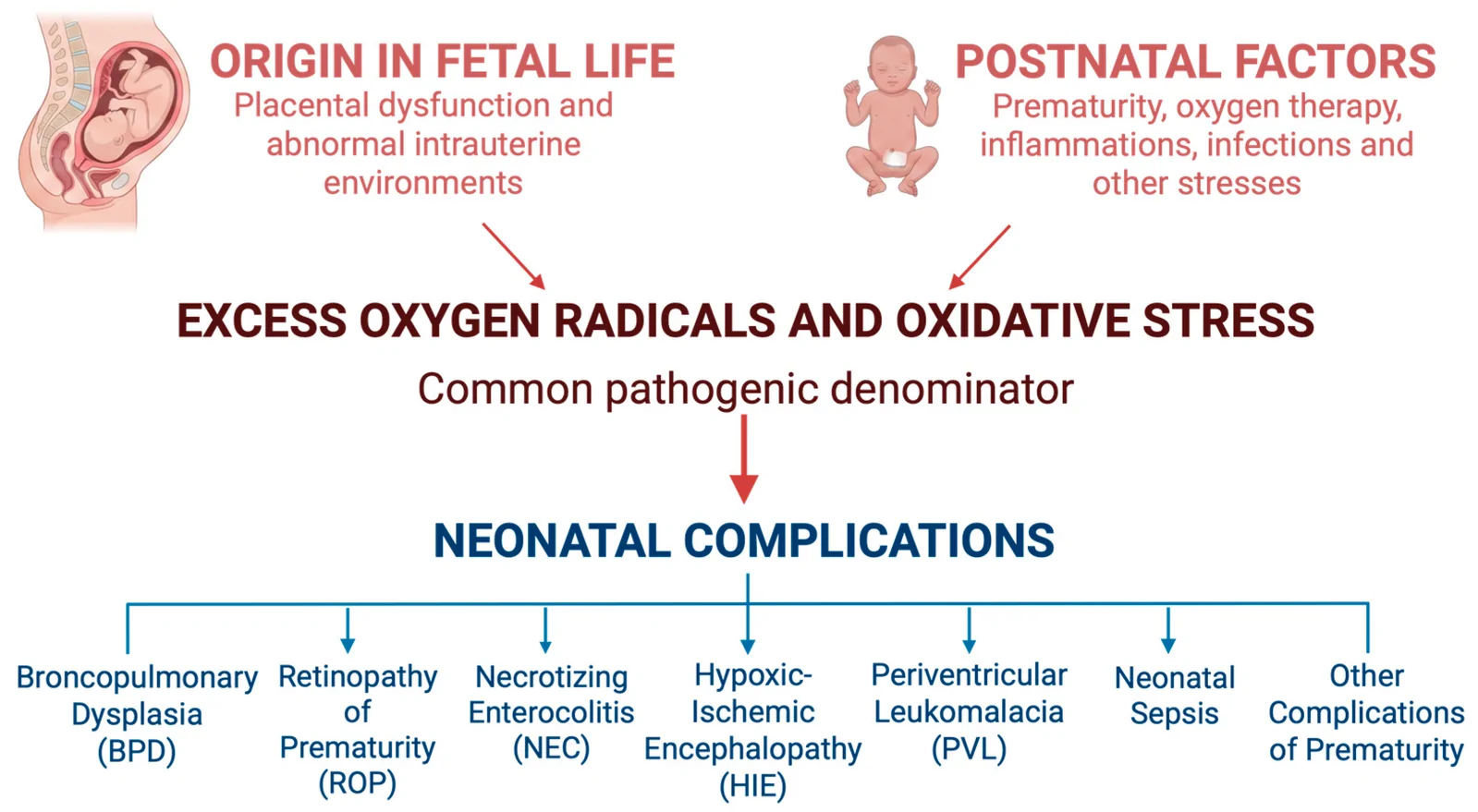
The oxygen radical diseases of neonatology. Created in BioRender. Mattei, A. (2026) https://BioRender.com/bmyjbe5.

Based on these observations, Saugstad first introduced the concept of the “oxygen radical diseases of neonatology” to describe a spectrum of neonatal disorders sharing oxidative stress as a common pathogenic denominator [6]. This paradigm included bronchopulmonary dysplasia (BPD), retinopathy of prematurity (ROP), necrotizing enterocolitis (NEC), hypoxic–ischemic encephalopathy (HIE), periventricular leukomalacia (PVL), neonatal sepsis, and other complications of prematurity. Increasing evidence now suggests that OS should not be considered exclusively a postnatal phenomenon, but rather a process originating during fetal life through placental dysfunction and abnormal intrauterine environments [7] [Figure 1]. In recent years, growing interest has focused on antioxidant therapies aimed at interrupting this continuum and limiting oxidative damage before irreversible cellular injury occurs. Among candidate molecules, melatonin has emerged as a particularly attractive therapeutic agent because of its pleiotropic biological properties and favorable safety profile [8,9]. Melatonin (*N*-acetyl-5-methoxytryptamine) is an endogenous indoleamine primarily synthesized by the pineal gland. It has been traditionally recognized as the principal regulator of circadian rhythms, but it also exerts powerful antioxidant, anti-inflammatory, antiapoptotic, and mitochondrial protective actions [8].

Importantly, melatonin is physiologically involved in pregnancy and fetal development. Maternal melatonin crosses the placenta and contributes to fetal circadian entrainment, neurodevelopment, and antioxidant protection throughout gestation. Given these pleiotropic properties, melatonin has been proposed as a potential multitarget strategy for modulating oxidative and inflammatory pathways across different stages of the perinatal period. However, whether these mechanistic effects translate into meaningful improvements in maternal, fetal, or neonatal clinical outcomes remains uncertain [8,10,11].

On the other hand, ROS should not be considered exclusively pathological. Physiological redox signaling is essential for normal development and cellular homeostasis, and controlled ROS production contributes to trophoblast function, angiogenesis, vascular oxygen sensing, cellular differentiation, and immune signaling [12,13,14]. Therefore, although excessive oxidative stress is implicated in neonatal tissue injury, indiscriminate or prolonged suppression of ROS could theoretically interfere with physiological redox signaling, particularly during critical developmental windows. However, direct evidence that melatonin disrupts physiological developmental or immune signaling in human neonates is currently lacking, and this possibility remains an important area for future investigation.

The aim of this narrative review is to critically examine current evidence regarding the role of melatonin as a transversal modulator of oxidative stress across fetal, perinatal, and neonatal life.

### 1.2. Methodology

This narrative review was informed by a non-systematic literature search of PubMed, Scopus, Web of Science and Cochrane Librar for articles published up to 2026, using combinations of the terms “melatonin,” “oxidative stress,” “neonatal,” “perinatal,” “placenta,” “hypoxic-ischemic encephalopathy,” “bronchopulmonary dysplasia,” “necrotizing enterocolitis,” “neonatal sepsis,” and “retinopathy of prematurity.” Preclinical and clinical studies, systematic reviews, and relevant clinical trial registrations were considered for inclusion. Non-English-language publications and abstract-only reports were generally excluded. As this is a narrative rather than a systematic review, no formal quality appraisal or risk-of-bias assessment was performed, and article selection and synthesis relied on the authors’ clinical and scientific judgment regarding relevance and methodological quality.

## 2. Why Is Melatonin Different from Other Antioxidants?

### 2.1. Melatonin and Its Pleiotropic Effects

Melatonin is one of the most versatile endogenous cytoprotective molecules and displays several unique pharmacological and biological characteristics that distinguish it from conventional antioxidants. Unlike vitamin-based antioxidants or isolated free radical scavengers, melatonin acts through multiple complementary mechanisms targeting different stages of oxidative injury and cellular dysfunction [15].

Firstly, melatonin has an amphiphilic nature, being both lipophilic and hydrophilic. This characteristic enables rapid diffusion through cellular membranes and broad tissue distribution, as it can cross the blood–brain barrier and placental barrier. Such widespread bioavailability is particularly important in fetal and neonatal medicine, where oxidative damage frequently involves multiple organs simultaneously, especially the brain, lungs, intestine, and retina [15,16].

In addition, circadian rhythms influence immune responses, mitochondrial metabolism and endocrine signaling. Therefore, melatonin exerts a pleiotropic role beyond its effect on circadian rhythm regulation. During fetal life, maternal melatonin represents the primary circadian signal reaching the fetus through placental transfer. In preterm infants, disruption of physiological circadian rhythms may further contribute to OS and inflammatory dysregulation, suggesting another possible therapeutic role for melatonin beyond its direct antioxidant properties [15,17,18].

Melatonin has direct scavenger action as it directly scavenges a wide range of ROS and RNS. Not only melatonin, but also its metabolites—cyclic-3-hydroxymelatonin, N1-acetyl-N2-formyl-5-methoxykynuramine, and N1-acetyl-5-methoxykynuramine—can neutralize FR through successive dehydrogenation reactions, creating a continuous free radical scavenging cascade. This sequential scavenging cascade may substantially amplify the overall antioxidant capacity of melatonin [16,19,20].

Beyond direct FR neutralization, melatonin enhances endogenous antioxidant defenses by upregulating antioxidant enzymes such as superoxide dismutase (SOD), glutathione peroxidase (GPx), glutathione reductase (GR) and catalase (CAT). Simultaneously, melatonin downregulates pro-oxidant enzymes including nitric oxide synthase (NOS) and lipoxygenases (LOS), thereby reducing further ROS generation [20].

In addition, melatonin exerts a role in cytoprotection through mitochondrial preservation. Indeed, mitochondria are both major sources and targets of ROS during hypoxic–ischemic and inflammatory injury. Melatonin accumulates within mitochondria, where it stabilizes mitochondrial membranes, improves electron transport chain efficiency, preserves adenosine triphosphate (ATP) production, and prevents opening of the mitochondrial permeability transition pore. Comprehensively, melatonin interrupts the vicious cycle linking mitochondrial dysfunction to further OS amplification [16,21]. Furthermore, melatonin acts as antiapoptotic by regulating mitochondrial apoptotic pathways, modulating Bcl-2 family proteins, inhibiting cytochrome-c release, and reducing caspase activation. Emerging evidence further suggests involvement in autophagy regulation, ferroptosis modulation, and epigenetic control of oxidative stress-related genes [15,20].

Melatonin has been reported to chelate redox-active transition metals, particularly copper and, to a lesser extent, iron, thereby limiting metal-catalyzed ROS generation. This metal-chelating property complements its direct radical-scavenging activity and mitochondrial protective effects, contributing to the overall antioxidant action of this molecule [21,22].

Beyond these receptor-independent effects, melatonin also acts through the membrane receptors MT1 and MT2, which are G-protein-coupled receptors expressed in several tissues. Their activation predominantly involves Gi proteins and inhibition of adenylyl cyclase, thereby modulating intracellular cAMP/PKA signaling. Through these receptor-dependent mechanisms, melatonin can influence cellular differentiation, survival, metabolism, and inflammatory responses [23]. Additional intracellular pathways, including Nrf2 and PI3K/Akt, have been implicated in melatonin-mediated cytoprotection and redox regulation, although their relative contribution appears to be context-dependent and is not equally established across tissues [24,25].

Finally, melatonin exerts potent anti-inflammatory effects through different mechanisms. It modulates gene expression and activities of NOS and cyclo-oxigenase and decreases the production of pro-inflammatory molecules [18]. In particular, melatonin may act as an immunological buffer, supporting immune responses when they are insufficient while limiting excessive inflammatory activation. These effects are mediated, at least in part, through MT1 and MT2 receptors expressed on immune cells, including T lymphocytes and natural killer cells, as well as through modulation of intracellular inflammatory signaling pathways [26,27]. Moreover, melatonin has been shown to inhibit NF-κB activation and consequently modulate the production of pro-inflammatory mediators, including TNF-α, IL-1β, and IL-6. Melatonin may also inhibit NLRP3 inflammasome activation, potentially through the reduction in mitochondrial ROS and preservation of mitochondrial homeostasis, thereby linking its anti-inflammatory effects to its antioxidant and mitochondrial protective activities [27,28]. An additional mechanism that may contribute to these effects involves the aryl hydrocarbon receptor (AhR). Experimental evidence indicates that melatonin and some of its metabolites can interact with the ligand-binding domain of AhR, promoting receptor activation and nuclear translocation and modulating the transcription of downstream target genes. Although the physiological relevance of this pathway in perinatal disease remains to be established, AhR signaling may represent an additional mechanism through which melatonin influences cellular antioxidant and protective responses [29].

**Figure 2:**
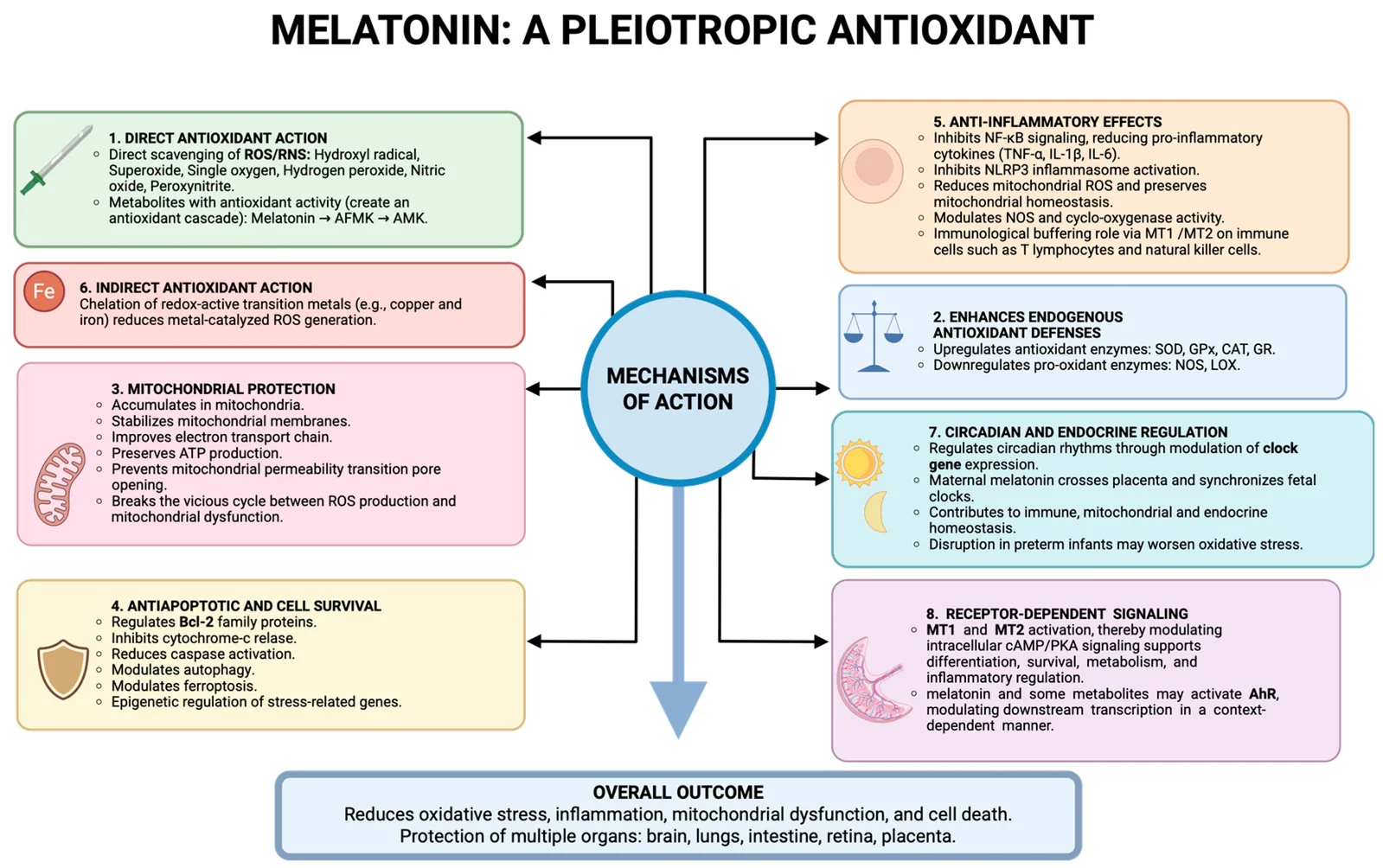
The pleiotropic effect of melatonin and its possible mechanisms of action as a cytoprotective and antioxidant molecule. Created in BioRender. Mattei, A. (2026) https://BioRender.com/zw9f3ld.

Collectively, these pleiotropic actions support the concept that melatonin should not be considered merely a conventional antioxidant molecule, but rather a multifunctional regulator of cellular homeostasis capable of simultaneously targeting oxidative stress, inflammation, mitochondrial dysfunction, and programmed cell death. This integrated biological activity may explain the broad protective effects observed across multiple models of placental, fetal, and neonatal disease [Figure 2].

Last but not least, melatonin appears to have a highly favorable safety profile. Experimental and clinical studies have generally reported minimal toxicity even at relatively high doses, a critical advantage when considering therapeutic applications in vulnerable populations such as preterm infants and neonates with critical illness. Unlike several antioxidant compounds, melatonin does not exhibit pro-oxidant effects at elevated concentrations and does not interfere negatively with physiological cellular metabolism [4,16]. However, the available safety data remains limited, particularly regarding prolonged exposure and long-term developmental outcomes.

### 2.2. Melatonin Compared with Other Conventional Antioxidant Therapies

Melatonin has attracted increasing interest as a potential neuroprotective agent in neonatal medicine because of its broad antioxidant and cytoprotective properties, which distinguish its pharmacological profile from that of conventional antioxidant approaches.

Vitamin C and vitamin E are non-enzymatic antioxidants that primarily provide hydrophilic and lipophilic redox protection, respectively. In newborn rats, intraventricular ascorbic acid significantly reduced the extent of hemispheric hypoxic–ischemic brain injury and was associated with reduced fodrin breakdown and necrotic cell death [30]. Similarly, in a ferret organotypic brain-slice model, vitamin E reduced cytotoxicity, preserved intracellular glutathione, and attenuated oxidative and inflammatory markers, including CHAC1, SLC7A11, TNF-α, and IL-8 [31]. However, the clinical neuroprotective efficacy of either vitamin in human neonates with HIE remains unestablished [32].

*N*-acetylcysteine (NAC) primarily supports endogenous antioxidant defenses through glutathione synthesis. In a neonatal rat model of hypoxic–ischemic injury, NAC improved several long-term sensorimotor outcomes [33]. In an early-phase clinical study of neonates with moderate-to-severe HIE undergoing therapeutic hypothermia, NAC combined with calcitriol produced dose-responsive increases in basal-ganglia glutathione and total creatine, including an approximately 32% increase in CNS glutathione within 30 min at 40 mg/kg, together with reduced plasma isofurans [34]. Although favorable neurodevelopmental outcomes were reported, the open-label, non-randomized design and concomitant calcitriol administration prevent determination of the independent contribution of NAC.

Long-chain omega-3 polyunsaturated fatty acids (LC-PUFAs), particularly DHA and EPA, may modulate membrane signaling, inflammation, lipid peroxidation, and specialized pro-resolving mediators. In neonates with HIE undergoing therapeutic hypothermia, circulating omega-3 levels were lower in infants with substantial brain injury, although this association does not establish causality or therapeutic benefit [35]. Their clinical efficacy and optimal dosing therefore remain uncertain.

Overall, melatonin has a distinctive pharmacological profile, combining amphiphilic distribution and direct scavenging of reactive oxygen and nitrogen species with potential effects on mitochondrial function, endogenous antioxidant defenses, inflammation, apoptosis, and receptor-mediated signaling [36]. However, these mechanistic properties do not establish clinical superiority. Direct head-to-head trials comparing melatonin with vitamin C, vitamin E, NAC, or omega-3 fatty acids are lacking, while differences in dose, timing, route of administration, formulation, and disease severity limit comparisons across studies. Therefore, the potential advantages of melatonin currently provide a rationale for further investigation rather than evidence of an established clinical advantage.

## 3. Melatonin and Placental Oxidative Stress

### 3.1. Placental Oxidative Stress and Pregnancy Complications

The proper development of the placenta is essential for the physiological development of the gestation. The progression of placental formation implies a fine balance between trophoblastic maturation, differentiation and apoptosis which leads to accelerated cellular turnover and excess ROS production. This increased OS, if not adequately counterbalanced, causes endothelial dysfunction and increased apoptosis of trophoblastic cells [37].

An abnormal placentation leads to impaired oxygen supply to the fetus which can contribute to several obstetric disorders, including preeclampsia (PE), gestational diabetes mellitus (GDM), intrauterine growth restriction (IUGR) and PTB [38,39].

Indeed, in PE, the abnormal placental implantation leads to decreased placental perfusion with consequent OS within the placenta. The consequent oxidative damage leads to inflammation; cellular death; and release of circulating factors, proinflammatory cytokines and vasoactive molecules. The cumulative result is a systemic inflammation which causes maternal hypertension and proteinuria, possible evolution into hepatic complications, disseminated vascular coagulation and brain problems with lethal outcomes. On the other hand, the reduced placental perfusion results in IUGR [40].

OS has also been implicated in several obstetric pathologies other than PE. IUGR can be caused by different pathologies, including maternal malnutrition, placental insufficiency or GDM, but animal models showed that all the etiologies of IUGR could potentially be linked to placental OS [40]. Similarly, in GDM there is a chronic placental exposure to hyperglycemia which has been linked to increased ROS production and oxidative damage [41]. Finally, PTB itself has been related to increased OS in several studies which reported higher levels of OS markers in preterm newborns compared to full-term ones. In preterm neonates, the immaturity of antioxidative defences cannot counterbalance the OS caused by placental hypoperfusion and by neonatal resuscitation care and this can lead to long-term consequences even when the baby survives [42].

### 3.2. Melatonin as Placental Protective Strategy

Given its antioxidant and anti-inflammatory properties, melatonin has emerged as a potential candidate for placental protection. Experimental studies suggest that melatonin may improve placental antioxidant capacity, reduce lipid peroxidation, preserve mitochondrial function, and attenuate inflammatory activation in models of placental insufficiency and fetal hypoxia [10,20].

It has been observed that maternal melatonin concentrations progressively increase throughout gestation, reaching peak levels during the third trimester, and rapidly decline after delivery. This physiological rise reflects both enhanced maternal pineal secretion and local placental and ovarian synthesis [18,43]. Indeed, melatonin is produced by both the oocyte and cells surrounding it, as it may contribute to protecting the oocyte from OS during maturation and ovulation and then it may support subsequent implantation [18,44].

After implantation, trophoblastic cells express both the enzymes required for melatonin biosynthesis and melatonin membrane receptors (MT1 and MT2), suggesting the existence of autocrine and paracrine regulatory pathways within placental tissue. Placental melatonin is produced at daily rhythmic intervals and freely crosses the placenta to reach the fetal circulation, where it exerts its role as a circadian regulator and antioxidant protector for the developing fetus throughout gestation [18,45].

From one perspective, melatonin influences epigenetic expression of fetal genes with a circadian rhythm. Consequently, circadian disruption occurring in pregnant women due to shift work, jet lag or a wrong daily routine can potentially affect fetal development and may have consequences in post-natal life [46,47].

On the other hand, melatonin plays a protective role through the neutralization of excessive ROS production which can occur at different steps of placentation. It contributes to maintaining a finely balanced redox equilibrium by inducing expression and activity of SOD and CAT, acting as a direct ROS scavenger and modulating molecular pathways which are linked to hypoxia-induced autophagy [39].

It is also capable of modulating placental angiogenesis and vascular function through interactions with nitric oxide pathways and endothelial signaling [47]. The action of melatonin in reducing chronic intrauterine OS may have a potential impact on long-term epigenetic and metabolic alterations associated with neurodevelopmental impairment, cardiovascular disease, and metabolic dysfunction later in life [18,48,49].

At the molecular level, melatonin’s placental actions are mediated through both receptor-dependent and receptor-independent pathways. Receptor-dependent effects occur mainly via membrane MT1 and MT2 receptors expressed on trophoblastic cells, whereas receptor-independent effects include direct free-radical scavenging and mitochondrial antioxidant actions that do not require receptor binding. Human placental trophoblasts express MT1 and MT2 receptors, and melatonin signaling has been implicated in trophoblast differentiation and protection against oxidative and hypoxia/reoxygenation-induced injury. Melatonin also activates endogenous antioxidant responses in placental tissue, including pathways involving Nrf2-associated antioxidant genes, although the precise relationship between receptor signaling and these antioxidant effects remains incompletely defined. Through these convergent pathways, melatonin may support trophoblast viability, limit oxidative and apoptotic injury, and contribute to placental adaptation; however, much of the mechanistic evidence remains derived from cell culture, animal models, or extrapolation from non-placental systems, and direct confirmation of specific signaling mechanisms in human placental tissue remains limited [47,50,51,52,53].

In addition to its direct antioxidant actions, melatonin may contribute to immune regulation at the maternal–fetal interface by modulating macrophage, NK-cell, and T-cell activity. Alterations in decidual NK cells, regulatory T cells (Tregs), Th17 cells, and the Th1/Th2 balance have been associated with pregnancy complications including preeclampsia. Experimental and review evidence suggests that melatonin can influence these immune populations and cytokine responses during pregnancy, although its precise effects on decidual macrophage polarization and the Treg/Th17 balance remain incompletely characterized. Recent evidence that human placental macrophages and trophoblasts express MT2 receptors and produce melatonin further supports a potential local role for melatonin in placental immune regulation. Nevertheless, evidence specifically demonstrating that melatonin directly alters maternal–fetal immune-cell populations in vivo remains preliminary, and many proposed mechanisms are based on broader immunological or non-placental studies [39,47]. Overall, current evidence supports the hypothesis that melatonin may act as a physiological and therapeutic regulator of placental redox homeostasis, potentially limiting the progression from placental OS to fetal and neonatal disease. This placental-fetal perspective further reinforces the rationale for considering melatonin as an integrated intervention across the entire perinatal OS continuum.

### 3.3. Antenatal Melatonin Administration for Fetal Protection

The increasing understanding of melatonin physiology during pregnancy has prompted growing interest in its therapeutic administration as a maternal intervention aimed at preventing fetal and neonatal complications associated with placental dysfunction and OS.

Different clinical trials investigated the use of melatonin as an antioxidant agent in different obstetric conditions. A randomized clinical trial (RCT) explored the efficacy of melatonin compared to quercetin antenatal supplementation to prevent neonatal complications, including hypoglycemia, PTB, birth injury, neonatal hyperbilirubinemia and respiratory distress, while demonstrating a favorable safety profile for both mother and fetus [54].

Melatonin has also shown preliminary promise in pregnancies complicated by PE. In a phase I clinical trial, Hobson et al. reported that maternal melatonin administration prolonged the interval from diagnosis to delivery, reduced the need for antihypertensive medications, and improved markers of endothelial function, supporting its potential role as an adjunctive therapy in early-onset PE [55]. Similarly, Miller et al. investigated antenatal melatonin administration in a small cohort of pregnancies complicated by FGR. Placental analysis demonstrated reduced OS and enhanced antioxidant capacity following maternal melatonin treatment, providing preliminary evidence that melatonin may directly modulate placental redox homeostasis in vivo [56].

However, robust RCTs evaluating long-term effects of antenatal melatonin on obstetric pathologies are still lacking.

From the fetal perspective, neuroprotection is one of the most promising potential clinical applications of antenatal melatonin. Indeed, OS and inflammation play important roles in the pathogenesis of preterm brain injury, including white matter damage and subsequent neurodevelopmental impairment. Hence, antenatal maternal melatonin administration has been proposed as a potential strategy to protect the developing fetal brain from hypoxic–ischemic injury, resulting in a safe antenatal intervention with the potential to improve long-term neurological outcomes in high-risk neonates [57].

However, to our knowledge, there is no available study in the literature evaluating the efficacy of antenatal melatonin administration on neuroprotection in preterm birth.

A multicentred PREMELIP trial was designed in 2018 (ClinicalTrials.gov identifier: NCT02395783) to assess the dose of melatonin to administer to pregnant women in preterm labour (<28 weeks of gestational age) in order to reduce brain damage in neonates born prematurely. However, the trial has ended, and no results have been provided yet [58].

Currently, a multicenter triple-blinded randomized controlled PROTECTME trial is ongoing (ClinicalTrials.gov identifier: NCT05651347) across Australia and New Zealand to determine the impact of antenatal melatonin supplementation on early childhood neurodevelopmental outcomes in patients with severe growth restriction. At the moment of publication, however, the trial is still in the recruiting phase.

Beyond neuroprotection, emerging clinical evidence suggests that maternal melatonin supplementation may also influence neonatal respiratory adaptation. In fact, antenatal melatonin administration in women at risk of preterm delivery has reported a significant reduction in the incidence of RDS, together with improved neonatal respiratory outcomes compared with the control group [59]. Although the underlying mechanisms remain to be fully elucidated, these potential beneficial effects are likely related to melatonin-mediated attenuation of OS and inflammation during the critical transition from intrauterine to extrauterine life, as well as to improved placental function and fetal tissue protection.

Finally, a growing body of preclinical evidence supports melatonin as a potential developmental reprogramming agent, capable of preventing the long-term consequences of adverse intrauterine environments. Animal studies have shown that maternal melatonin administration during pregnancy and lactation attenuates OS and reduces the offspring’s susceptibility to hypertension, chronic kidney disease, and metabolic syndrome later in life [60].

These protective effects appear to be mediated, at least in part, through epigenetic regulation of fetal organ development. In particular, melatonin has been shown to modulate gene expression during nephrogenesis, promoting normal kidney development and reducing the risk of programmed hypertension in adulthood [61]. Similarly, maternal melatonin supplementation has been reported to prevent cardiovascular dysfunction in adult offspring exposed to prenatal hypoxia, highlighting its potential ability to counteract fetal cardiovascular programming [62]. Beneficial metabolic effects have also been reported, with maternal melatonin improving energy metabolism, reducing insulin resistance and diabetes susceptibility, and preventing hepatic lipid alterations induced by maternal high-fat diet in the offspring [63,64].

Despite these compelling experimental findings, translation into clinical practice remains limited. To date, no human RCT has demonstrated that antenatal melatonin supplementation improves long-term cardiovascular, renal, or metabolic outcomes in offspring. Therefore, although current evidence supports maternal melatonin administration as a biologically plausible and potentially promising strategy to modulate placental OS and improve fetal and neonatal outcomes, adequately powered randomized controlled trials with long-term follow-up are still required to establish its efficacy, optimal dosing regimen, and safety before routine clinical use can be recommended.

## 4. Melatonin as a Postnatal Neuroprotective Strategy

### 4.1. Oxidative Stress and Hypoxic–Ischemic Encephalopathy

Perinatal hypoxic–ischemic injury remains one of the leading causes of neonatal mortality and long-term neurodevelopmental disability worldwide, with an incidence of approximately 2 every 1000 newborn in developed countries. In developing countries, however, these statistics can reach up to 20 every 1000 neonates [65]. HIE is associated with significant risks of cerebral palsy, epilepsy, cognitive impairment, and behavioural disorders. Despite the introduction of therapeutic hypothermia, which currently represents the standard of care for moderate-to-severe HIE, neurological outcomes remain suboptimal in a substantial proportion of affected neonates [66]. Consequently, considerable efforts have focused on identifying adjunctive neuroprotective strategies capable of targeting the multiple mechanisms involved in hypoxic–ischemic brain injury.

OS plays a pivotal role in the pathogenesis of neonatal hypoxic–ischemic brain damage. The initial hypoxic insult leads to primary energy failure characterized by ATP depletion, excitotoxicity, intracellular calcium overload, and impaired mitochondrial function. Excessive glutamate release and subsequent activation of excitatory receptors contribute to intracellular Ca^2+^ accumulation, further increasing mitochondrial dysfunction and cellular energy failure. Subsequently, a further component of tissue injury occurs during the subsequent reperfusion phase, when reoxygenation paradoxically triggers excessive production of ROS and RNS, which can initiate neuroinflammatory signaling and the release of pro-inflammatory cytokines, generating a self-amplifying cascade of neuronal and glial injury. Consequently, it can lead to neuronal toxicity, mitochondrial dysfunction, and cellular apoptosis [67].

The neonatal brain is particularly susceptible to oxidative injury because of several intrinsic characteristics, including high oxygen consumption, abundant free iron, immature antioxidant systems, and limited endogenous detoxification capacity. The initial FR release can occur within the first 30 min of asphyxia while the consequences of this cascade could occur during the following 28 days and potentially contribute to prolonged secondary brain injury [16].

### 4.2. Melatonin as a Brain Protective Strategy

Melatonin has emerged as one of the most promising neuroprotective agents in experimental models of neonatal hypoxia-ischemia.

Its protective effects extend far beyond simple FR scavenging and involve multiple complementary mechanisms targeting the complex pathophysiology of brain injury. As previously mentioned, melatonin preserves mitochondrial function through mitochondrial membrane stabilization, improves electron transport chain efficiency through mitochondrial ROS reduction, ATP synthesis preservation, and mitochondrial permeability decrease. Through these actions, melatonin attenuates secondary energy failure and limits neuronal cell death. In addition to its antioxidant and mitochondrial effects, melatonin may influence mechanisms directly involved in hypoxic–ischemic neuronal injury, including excitotoxicity and intracellular calcium dysregulation. By preserving mitochondrial homeostasis and cellular energy metabolism, melatonin may limit the downstream consequences of excessive Ca^2+^ accumulation and reduce activation of mitochondrial-dependent cell-death pathways [4,68,69,70].

In parallel, melatonin exerts potent anti-inflammatory effects within the injured brain. Experimental studies demonstrate reduced activation of microglia and astrocytes, decreased production of pro-inflammatory cytokines such as TNF-α, IL-1β, and IL-6, and suppression of NF-κB-mediated inflammatory signalling. Recent evidence also suggests that melatonin modulates microglial polarization, promoting a shift toward anti-inflammatory and reparative phenotypes [4,69,70]. In addition, melatonin modulates programmed cell death pathways; thus, it may impact brain cell preservation [4,69,70,71,72].

Experimental models consistently demonstrate significant histological and functional neuroprotection following melatonin administration. In animal models of perinatal asphyxia and neonatal hypoxia-ischemia, melatonin reduces cerebral infarct volume, attenuates white matter injury, decreases lipid peroxidation, and improves long-term neurobehavioral outcomes. Importantly, several experimental studies indicate that melatonin remains effective even when administered after the initial hypoxic insult, supporting its potential clinical applicability [4,68,70,72].

In human studies, the interaction between melatonin and therapeutic hypothermia (TH) has attracted particular interest. Since TH primarily reduces cerebral metabolism and excitotoxicity, whereas melatonin additionally targets OS, inflammation, and apoptosis, combined therapy may provide complementary and potentially synergistic neuroprotection. Experimental evidence supports enhanced efficacy of combined melatonin-hypothermia treatment compared with TH alone, with greater reductions in oxidative injury and improved neuronal survival [4,68,69,70]. However, whether these experimental findings translate into clinically meaningful additional benefit beyond therapeutic hypothermia remains to be established.

Furthermore, melatonin has generally shown a favourable safety profile in the limited clinical studies conducted to date, and it is relatively inexpensive and widely available, factors that may facilitate future implementation in low-resource settings where neonatal hypoxic–ischemic injury remains highly prevalent [9,69,70].

Nevertheless, evidence regarding long-term safety, particularly after repeated administration during critical periods of brain development, remains limited.

To date, a limited number of studies have explored the feasibility of melatonin administration in human HIE.

Fulia et al. conducted the first clinical study investigating the neuroprotective potential of melatonin in 10 asphyxiated newborns by measuring plasma malondialdehyde, a marker of lipid peroxidation, and nitrite/nitrate concentrations at 12 and 24 h after birth. Infants treated with melatonin showed a significant reduction in both oxidative stress markers, providing preliminary clinical evidence of its antioxidant efficacy in neonatal hypoxic injury [73].

Subsequently, Aly et al. performed a randomized controlled pilot study involving 30 neonates with HIE, demonstrating that the addition of melatonin to therapeutic hypothermia resulted in improved neurological outcomes compared with hypothermia alone. Specifically, melatonin-treated infants experienced fewer electrographic seizures and exhibited less white matter injury on neuroimaging [74].

These findings were further supported by El Farargy and Soliman, who reported that adjunctive melatonin therapy improved short-term survival in neonates with HIE. In the same randomized controlled trial, the combination of melatonin and magnesium sulphate significantly reduced serum concentrations of S100B, a well-established biomarker of brain injury, suggesting enhanced neuroprotection through attenuation of oxidative stress and neuronal damage [75].

More recently, a multicentred randomized pilot study by Jerez-Calero et al. evaluated the addition of melatonin to TH in 25 newborns with HIE. Infants receiving melatonin demonstrated improved cognitive outcomes at 18 months of age compared with those treated with hypothermia alone, supporting a potential long-term neuroprotective effect. A subsequent follow-up study from the same group showed that improved neurodevelopmental outcomes were associated with a more favourable profile of circulating pro-inflammatory biomarkers during the first 10 days of life, further suggesting that modulation of the inflammatory response contributes to melatonin-mediated neuroprotection [76].

Overall, clinical evidence supporting melatonin as an adjunctive therapy for neonatal HIE remains encouraging but preliminary. Most available studies are limited by small sample sizes, heterogeneous dosing regimens, timing and route of administration, and relatively short follow-up periods. Consequently, the optimal therapeutic window, dosage, target plasma concentrations, and long-term safety have yet to be established, particularly in preterm infants [77]. To address these limitations, a large multicentred RCT (ClinicalTrials.gov identifier: NCT02621944) is currently evaluating the efficacy of melatonin combined with TH on long-term neurodevelopmental outcomes in neonates with HIE, with study completion anticipated in 2027. The results of this and other adequately powered trials will be essential to determine whether the biological and experimental benefits of melatonin translate into clinically meaningful improvements in survival and neurodevelopment.

Beyond HIE, OS-mediated brain injury also contributes to other neurological complications of prematurity, including PVL, diffuse white matter injury, and impaired neurodevelopmental maturation. Since inflammation and oxidative imbalance frequently coexist in these conditions, melatonin may potentially exert broader neuroprotective effects across different forms of neonatal brain injury [72,78,79].

## 5. Melatonin as Lung Protective Strategy

### 5.1. Bronchopulmonary Dysplasia and Respiratory Oxidative Injury

BPD is the most common chronic lung disease of preterm neonates treated with oxygen and positive-pressure ventilation and one of the leading causes of long-term respiratory morbidity among very preterm infants. Despite advances in neonatal intensive care, BPD still affects approximately 30–40% of extremely preterm infants, with the incidence increasing as gestational age and birth weight decrease. BPD is characterized by arrested alveolar and pulmonary vascular development resulting from the interaction of prenatal and postnatal insults. Oxidative stress is recognized as a central mechanism in its pathogenesis. In particular, the immature antioxidant defences of preterm infants are overwhelmed by excessive reactive oxygen species generated by hyperoxia, mechanical ventilation, inflammation, infection, and intermittent hypoxia. The resulting oxidative imbalance activates inflammatory signalling, impairs angiogenesis and alveolarization, disrupts mitochondrial function, and ultimately leads to simplified alveolar architecture and abnormal pulmonary vascular development. Arrested alveolarization represents a central pathological feature of BPD, with fewer and larger alveoli and reduced gas-exchange surface area. In parallel, impaired pulmonary angiogenesis and abnormal pulmonary vascular development contribute to increased pulmonary vascular resistance and may predispose affected infants to pulmonary hypertension [80,81,82].

The immature lung is highly susceptible to oxidative injury because antioxidant enzymes such as SOD, CAT, and GPx are underdeveloped in preterm infants. Exposure to supplemental oxygen and ventilator-induced lung injury further amplifies ROS generation and inflammatory signalling [80,81].

### 5.2. Melatonin in BPD

Experimental studies suggest that melatonin may attenuate several pathogenic mechanisms involved in BPD development. Melatonin reduces lipid peroxidation, suppresses inflammatory cytokine production, limits neutrophil infiltration, and preserves mitochondrial function within pulmonary tissue. In animal models of hyperoxia-induced lung injury, melatonin administration improves alveolar architecture and reduces oxidative biomarkers. Beyond direct antioxidant effects, melatonin may potentially influence disease-specific mechanisms involved in BPD, including preservation of alveolar development, endothelial function, angiogenesis, and pulmonary vascular integrity. In addition to its antioxidant activity, melatonin may exert antifibrotic and vascular protective effects, potentially influencing long-term pulmonary remodelling [83,84].

To date there is a paucity of evidence on the use of melatonin as a proactive strategy in BDP neonates.

The first clinical studies by Gitto et al. demonstrated that preterm infants with RDS treated with melatonin exhibited significantly lower circulating levels of pro-inflammatory cytokines and oxidative stress markers, accompanied by improved respiratory parameters compared with control infants. A subsequent study by the same group further showed that melatonin reduced inflammatory cytokine levels and was associated with a lower incidence of BPD, suggesting that attenuation of pulmonary inflammation may contribute to improved respiratory outcomes. However, these studies were relatively small and primarily evaluated biochemical and short-term respiratory outcomes, limiting conclusions regarding the causal effect of melatonin on BPD development [85,86].

These findings were later supported by an RCT conducted by Elfarargy et al., including 100 mechanically ventilated preterm infants with RDS. Neonates receiving adjunctive melatonin therapy showed reduced systemic inflammatory markers, shorter duration of incubator stay, and a lower incidence of BPD compared with those receiving standard treatment alone. Although these results are encouraging, the relatively limited sample size and study-specific treatment protocol warrant confirmation in larger multicenter trials [87].

More recently, Gharehbaghi et al. evaluated the efficacy of melatonin for the prevention of BPD in 80 preterm infants with RDS treated with surfactant using the INSURE technique. Infants receiving enteral melatonin for three days experienced significantly lower mortality and shorter hospital stay than controls, further supporting the potential clinical benefits of melatonin in neonatal respiratory disease. Nevertheless, these findings should be interpreted cautiously, as mortality and length of hospital stay are not specific measures of BPD prevention, and larger studies are required to determine whether melatonin directly modifies the incidence or severity of BPD [84].

Overall, although the available clinical evidence is still limited by relatively small sample sizes and heterogeneous treatment protocols, these studies provide preliminary evidence that melatonin may modulate pulmonary oxidative and inflammatory pathways and may be associated with improved respiratory outcomes in preterm infants. However, a direct causal effect on BPD prevention has not yet been firmly established. Larger multicenter randomized controlled trials are needed to confirm these findings and establish the optimal dosing regimen, timing of administration, and long-term safety of melatonin in the prevention of BPD.

## 6. Melatonin and Gut Barrier Dysfunction

### 6.1. Necrotizing Enterocolitis and Oxidative Stress

NEC is among the most feared gastrointestinal emergencies in neonatology. It is estimated that approximately 7% of preterms with a body weight between 500 and 1500 g develop NEC. NEC is characterized by intestinal inflammation that can progress to mucosal injury, intestinal necrosis, systemic inflammation, sepsis, multiorgan dysfunction, and death. Its pathogenesis is multifactorial and involves an interplay between intestinal immaturity, impaired epithelial barrier function, ischemia–reperfusion injury, dysbiosis, exaggerated inflammatory responses, and oxidative stress. The immature intestine of preterm infants is particularly susceptible to injury because of an incompletely developed epithelial barrier, immature innate and adaptive immune defenses, and altered intestinal vascular development and tone. The intestinal barrier is further challenged by postnatal factors, including enteral feeding, antibiotic exposure, and other intensive care-related interventions, which can contribute to microbial dysbiosis and abnormal intestinal immune activation [88].

Increasing evidence has linked OS and NEC [89,90,91].

It has been speculated that the immaturity of the neonatal gut results in an exaggerated immune response against lipopolysaccharide presented by intestinal bacteria, potentially mediated by Toll-like receptor 4 (TLR4) signaling, with consequent enterocyte apoptosis followed by delayed injury repair [4,69,70]. These processes lead to increased ROS production, which contributes to disruption of intestinal barrier integrity, increased permeability, bacterial translocation, and amplification of inflammatory cascades. Mitochondrial dysfunction and impaired microcirculatory perfusion further exacerbate intestinal damage [91,92].

### 6.2. Melatonin as a Gut Protective Strategy

Melatonin seems to be particularly interesting in NEC because of its combined antioxidant, anti-inflammatory, and barrier-protective properties. Experimental studies demonstrate that melatonin preserves tight junction integrity, reduces intestinal permeability, attenuates lipid peroxidation, and suppresses inflammatory cytokines within intestinal tissue [93,94,95].

Although most evidence currently derives from experimental models, these findings support further exploration of melatonin as a possible adjunctive strategy in NEC prevention and treatment. However, the mechanistic evidence should be interpreted cautiously, as the extent to which these molecular effects translate into clinically meaningful protection in neonates remains uncertain.

Initial studies have primarily evaluated melatonin as an adjunctive therapy in neonates with established NEC. In a non-RCT, melatonin administration in 50 neonates with NEC was associated with significant improvements in both clinical and laboratory parameters compared with standard treatment alone [96]. Similar findings were reported by Gad Allah et al., who observed that the addition of melatonin to antibiotic therapy in 20 preterm infants with NEC resulted in favourable clinical outcomes and attenuation of biochemical markers of inflammation and OS [97]. Although these studies provide preliminary clinical evidence, their small sample sizes and non-randomized designs substantially limit the ability to establish a causal therapeutic effect.

More recently, Shaaban et al. evaluated the preventive role of enteral melatonin in an RCT involving 90 preterm infants. Compared with conventional management, melatonin supplementation significantly reduced the incidence of NEC, improved feeding tolerance, and shortened hospital stay, supporting its potential as a prophylactic intervention in high-risk preterm neonates [98].

Overall, although these findings are encouraging, the available evidence is based on a limited number of relatively small studies. Larger multicentred RCTs are needed to confirm the efficacy and safety of melatonin for NEC prevention and to establish the optimal dosing regimen and timing of administration. An ongoing RCT (ClinicalTrials.gov identifier: NCT05033639) is currently evaluating the preventive role of melatonin in preterm infants, although results have not yet been published.

## 7. Melatonin and Neonatal Sepsis

### 7.1. Neonatal Sepsis and Immune Dysregulation

Neonatal sepsis is a complex systemic disorder resulting from the interaction between invading pathogens and an immature and dysregulated host response. Preterm infants are especially susceptible because of impaired pathogen containment, reduced phagocytic and chemotactic activity, immature adaptive immune responses, and limited antioxidant capacity. These features may facilitate systemic dissemination of pathogens and contribute to the development of a dysregulated inflammatory response, particularly in the most immature infants [99,100,101].

The inflammatory response represents a central component of neonatal sepsis. Following systemic pathogen dissemination, activation of innate immune pathways induces the release of inflammatory mediators and activation of endothelial cells. However, the neonatal inflammatory response is not simply characterized by excessive inflammation, but rather by a complex, developmentally regulated dysregulation of immune responses. In particular, IL-6 and IL-8 have been identified as important mediators of the neonatal sepsis cascade, although the cytokine profile may vary according to gestational age, pathogen, and timing of sepsis onset [99,100,101].

The vascular endothelium represents another critical component of neonatal sepsis pathophysiology. Endothelial cells interact with pathogens, circulating immune cells, platelets, and inflammatory mediators and contribute to the regulation of vascular permeability, leukocyte recruitment, coagulation, and microvascular perfusion. During sepsis, endothelial activation and injury may lead to increased vascular permeability, impaired vascular reactivity, and heterogeneous tissue perfusion. These alterations may contribute to hypotension, tissue hypoxia, and ultimately multiorgan dysfunction. Importantly, the neonatal endothelium has a limited capacity to counteract oxidative injury, potentially contributing to the distinct vascular response observed in septic neonates [99,100,101].

Oxidative stress represents an important downstream component of this inflammatory and vascular response. The activation of inflammatory pathways promotes the production of ROS and RNS, while mitochondrial dysfunction may further increase intracellular ROS generation. In neonatal sepsis, both pro-oxidant pathways and endogenous antioxidant defenses are activated; however, the overall redox balance may be shifted toward oxidative stress, as demonstrated by increased markers of oxidative damage [99,100,101].

Thus, neonatal sepsis may be viewed as the result of interconnected immune, endothelial, microcirculatory, and redox disturbances rather than as an isolated infectious process. Thus, neonatal sepsis may be viewed as the result of interconnected immune, endothelial, microcirculatory, mitochondrial, and redox disturbances rather than as an isolated infectious process. This has led to growing interest in antioxidant strategies, such as melatonin, as potential adjuvant strategies for neonatal sepsis [99,100,101].

### 7.2. Melatonin as Protective Strategy

The role of melatonin in neonatal sepsis has been investigated in a few clinical trials.

One of the earliest clinical investigations was conducted by Gitto et al. in 2001, demonstrating that melatonin administration in septic neonates significantly reduced circulating markers of OS, providing the first evidence of its potential therapeutic benefit in neonatal sepsis [102].

Subsequent clinical studies have reported potentially favourable biochemical and clinical effects of adjunctive melatonin therapy. El-Gendy et al. reported that melatonin administration as an adjunct to conventional treatment was associated with improvements in clinical and laboratory parameters in neonates with sepsis. Their prospective, non-randomized, non-blinded case–control study included 40 neonates with neonatal sepsis [103].

In a subsequent randomized controlled trial, El-Kabbany et al. evaluated 40 preterm infants with neonatal sepsis who were randomly assigned to receive either melatonin in addition to conventional therapy or conventional therapy alone. Melatonin-treated infants showed more favourable clinical and laboratory outcomes, including lower inflammatory markers and reduced malondialdehyde levels, compared with controls. Mortality was also lower in the melatonin group, although the small sample size limits the strength and generalizability of these findings [104].

Despite promising preliminary findings, current evidence remains insufficient to support routine clinical use. Larger controlled trials are needed to determine whether melatonin may improve clinically meaningful outcomes such as mortality, organ dysfunction, or neurodevelopmental prognosis.

## 8. Melatonin and Retinopathy of Prematurity

ROP is another classical OS-mediated complication of prematurity. Fluctuating oxygen exposure disrupts physiological retinal vascular development and promotes abnormal neovascularization through oxidative and inflammatory mechanisms. The immature retina is particularly vulnerable to oxidative injury because of its high metabolic demand, ongoing vascular development, and limited antioxidant capacity. Following preterm birth, exposure to the relatively hyperoxic extrauterine environment suppresses Hypoxia-Induced Factor 1α (HIF-1α) and VEGF signaling, leading to arrested physiological retinal vascularization and, in some cases, regression of immature vessels. As retinal maturation progresses, the metabolic demand of the avascular retina increases, resulting in relative tissue hypoxia. This hypoxic environment stabilizes HIF-1α and upregulates VEGF and other proangiogenic mediators, ultimately driving pathological retinal neovascularization. Throughout both phases, excessive ROS, mitochondrial dysfunction, and inflammatory signaling contribute to retinal neurovascular injury and disease progression [105,106,107].

Experimental studies suggest that melatonin may attenuate retinal oxidative injury through direct FR scavenging, mitochondrial protection, and modulation of inflammatory pathways. Melatonin receptors are also expressed within retinal tissue, suggesting potential physiological roles in retinal development and photoreceptor protection [108,109].

However, clinical evidence regarding melatonin use in ROP prevention remains limited, and further translational studies are required.

Table 1 summarizes the proposed melatonin mechanisms, reported effects, and route of administration across perinatal oxidative stress-related conditions discussed in this review.

## 9. Pharmacokinetics of Melatonin in Preterms

The pharmacokinetic profile of melatonin in newborns differs markedly from that observed in adults, primarily because of the developmental immaturity of hepatic and renal clearance mechanisms. In preterm neonates, oral administration of melatonin at doses ranging from 0.5 to 5 mg/kg has been associated with rapid absorption, high peak plasma concentrations, and a markedly prolonged elimination half-life of approximately 8–15.8 h, considerably longer than the 30–90 min typically reported in adults [110,111]. The prolonged half-life may reflect the immature CYP1A2 activity, the principal enzyme responsible for melatonin hydroxylation, together with immature phase II conjugation pathways and reduced renal excretory capacity in premature infants [110]. These developmental factors may contribute to higher circulating concentrations and greater systemic exposure compared with adults receiving equivalent weight-adjusted doses.

Additional pharmacokinetic data are available in neonates with HIE undergoing TH. In a small study of five neonates, enteral administration of melatonin at 0.5 mg/kg during hypothermia resulted in a peak plasma concentration of approximately 0.25 μg/mL and a 24 h AUC of 4.35 μg/mL·h. Melatonin clearance and elimination were prolonged compared with adult values, while pharmacokinetic parameters did not appear to be substantially altered by therapeutic hypothermia. However, these findings should be interpreted cautiously given the very small sample size [112].

Despite these observations, several clinically relevant pharmacokinetic questions remain unresolved. In particular, data comparing oral, enteral, and intravenous administration are scarce, and the absolute and relative bioavailability of melatonin in neonates remains insufficiently characterized. Moreover, the extent to which pharmacokinetic parameters differ between preterm and term neonates, and how gestational and postnatal age influence melatonin clearance and systemic exposure, remains unclear. Importantly, although preliminary data suggest that therapeutic hypothermia may not substantially modify melatonin pharmacokinetics, this observation requires confirmation in larger cohorts. Finally, no validated target plasma concentration or established pharmacokinetic–pharmacodynamic relationship has yet been defined for neonatal neuroprotection. Thus, the prolonged systemic exposure observed in neonates should not necessarily be interpreted as evidence of therapeutic efficacy, and further pharmacokinetic studies are needed to establish appropriate dosing strategies and exposure targets [10,74,110,111].

## 10. Conclusions

Despite growing experimental and clinical evidence supporting the antioxidant and cytoprotective effects of melatonin and its safety, several issues still limit its translation into routine antenatal and neonatal practice. A major challenge is the marked heterogeneity of existing studies in terms of design, populations, dosing regimens, timing, and routes of administration, which prevents the development of standardized protocols.

Timing of administration is another key issue, as oxidative injury evolves dynamically across prenatal and postnatal phases. Consequently, melatonin effects may vary depending on whether it is administered prenatally, during acute hypoxic–ischemic injury, or in later inflammatory and reparative phases.

Although generally considered safe, robust long-term safety data in extremely preterm infants are still lacking, particularly regarding neurodevelopmental and endocrine outcomes. In addition, because reactive oxygen species also play essential physiological roles, excessive antioxidant therapy may theoretically disrupt normal redox signalling.

Melatonin’s appeal lies in its pleiotropic activity, extending beyond direct radical scavenging to include modulation of mitochondrial function, inflammation, apoptosis, and circadian biology. Emerging evidence also suggests interactions with ferroptosis, inflammasome activation, and microglial polarization, reinforcing its role as a systems-level regulator.

Future research should focus on large, standardized randomized controlled trials with long-term follow-up, alongside precision medicine approaches incorporating biomarkers and pharmacokinetic profiling to identify responders. Combination strategies with hypothermia, erythropoietin, or microbiota-targeted therapies may further enhance efficacy.

Overall, while the biological rationale is strong, clinical implementation of melatonin in neonatology will require rigorous translational studies to define optimal dosing, timing, and patient selection.

## Figures and Tables

**Table 1:** Summary of proposed melatonin mechanisms, reported effects, and route of administration across perinatal oxidative stress-related conditions discussed in this review. Level of evidence reflects the study designs cited in the corresponding sections (preclinical/small non-randomized or pilot clinical studies/randomized controlled trials).

Condition	Main Proposed Mechanism(s) of Melatonin Action	Reported Effects (As Cited in Text)	Route/Regimen Reported	Level of Evidence
Placental OS/PE, FGR	Antioxidant (SOD/CAT induction), MT1/MT2-mediated signaling, anti-inflammatory, modulation of angiogenesis	Prolonged interval to delivery and improved endothelial markers in early-onset PE (Hobson et al. [55]); reduced placental OS and enhanced antioxidant capacity in FGR (Miller et al. [56])	Oral, trial-specific dosing	Phase I trial/small cohort study
HIE	Mitochondrial protection, anti-excitotoxic, anti-apoptotic, anti-inflammatory (NF-κB ↓)	Reduced OS biomarkers (Fulia et al. [73]); improved neurological outcomes and reduced S100B as adjunct to hypothermia (Aly et al.; El Farargy & Soliman; Jerez-Calero et al. [78,79,80])	IV/oral, adjunct to therapeutic hypothermia	Randomized pilot studies (*n* = 10–30)
BPD	Antioxidant, anti-inflammatory, preservation of alveolar and vascular development	Lower cytokines and OS markers, reduced BPD incidence (Gitto et al. [89,90]); shorter incubator stay (Elfarargy et al., *n* = 100 RCT [87]); lower mortality (Gharehbaghi et al., *n* = 80 [84])	Enteral, short courses (e.g., 3 days)	RCTs, small-to-moderate sample size
NEC	Tight-junction preservation, anti-inflammatory, possible TLR4 modulation	Improved clinical/laboratory parameters (Elfrargy & Soliman, *n* = 50 non-RCT [96]; Gad Allah et al., *n* = 20 [97]); reduced NEC incidence (Shaaban et al., *n* = 90 RCT [98])	Enteral, adjunct to standard therapy	Mostly small non-RCT/one RCT
Neonatal sepsis	Antioxidant, endothelial protection, immune modulation	Reduced OS markers (Gitto et al. [102]); improved clinical/laboratory outcomes (El-Gendy et al., *n* = 40 non-RCT [103]; El-Kabbany et al., *n* = 40 RCT [104])	IV/oral, adjunct to antibiotics	Small RCT/non-RCT
ROP	Antioxidant, mitochondrial protection, retinal receptor-mediated signaling	Preclinical evidence only; no clinical trials identified	Not established in humans	Preclinical/experimental only

## Data Availability

No new data were created or analyzed in this study. Data sharing is not applicable to this article.
